# Prevalence of *Plasmodium falciparum* isolates lacking the histidine rich protein 2 gene among symptomatic malaria patients in Kwilu Province of the Democratic Republic of Congo

**DOI:** 10.1186/s40249-021-00860-1

**Published:** 2021-05-25

**Authors:** Yannick Bazitama Munyeku, Alain Abera Musaka, Medard Ernest, Chris Smith, Paul Mankadi Mansiangi, Richard Culleton

**Affiliations:** 1 Direction Des Laboratoires de Santé, Ministère de La Santé, Kinshasa, Democratic Republic of the Congo; 2 Institut National de Recherche Biomédicale (INRB), Laboratoire de Virologie Clinique, Kinshasa, Democratic Republic of the Congo; 3 Graduate School of Tropical Medicine and Global Health (TMGH), Nagasaki University, Nagasaki, Japan; 4 Malaria Unit, Department of Pathology, Institute of Tropical Medicine, Nagasaki University, Nagasaki, Japan; 5 Division Provinciale de La Santé du Kwilu, Kwilu, Democratic Republic of the Congo; 6 Kinshasa School of Public Health, Faculty of Medicine, University of Kinshasa, Kinshasa, Democratic Republic of the Congo; 7 Laboratory of Malaria and Vector Research, National Institute of Allergy and Infectious Diseases, National Institutes of Health, Rockville, MD 20852 USA; 8 Department of Clinical Research, London School of Hygiene & Tropical Medicine, London, UK; 9 Institut Supérieur Des Techniques Médicales (ISTM), Kikwit, DR Congo; 10 Department of Protozoology, Institute of Tropical Medicine (NEKKEN), Nagasaki, Japan; 11 Division of Molecular Parasitology, Proteo-Science Centre, Ehime University, Toon, Japan

**Keywords:** Plasmodium *falciparum* histidine rich protein 2, Gene deletion, False negative, Rapid diagnostic test, Symptomatic patient

## Abstract

**Background:**

Malaria rapid diagnostic tests have become a primary and critical tool for malaria diagnosis in malaria-endemic countries where *Plasmodium falciparum* Histidine Rich Protein 2-based rapid diagnostic tests (*Pf*HRP2-based RDTs) are widely used. However, in the last decade, the accuracy of *Pf*HRP2-based RDTs has been challenged by the emergence of *P. falciparum* strains harbouring deletions of the *P. falciparum histidine rich protein 2* (*pf*hrp2) gene, resulting in false-negative results. In the Democratic Republic of Congo (D.R. Congo), little is known about the prevalence of the *pfhrp2* gene deletion among *P. falciparum* isolates infecting symptomatic patients, especially in low to moderate transmission areas where *pfhrp2* deletion parasites are assumed to emerge and spread. Here we determine the local prevalence and factors associated with *pfhrp2* gene deletions among symptomatic malaria patients in the Kwilu Province of the D.R. Congo.

**Methods:**

We used secondary data from a prospective health facility-based cross-sectional study conducted in 2018. Blood was collected for microscopy, *Pf*HRP2-RDT, and spotted onto Whatman filter paper for downstream genetic analysis. Genomic DNA was extracted and used to perform PCR assays for the detection and confirmation of *pf*hrp2 gene deletions. Fischer’s exact and the Kruskal–Wallis tests were applied to look for associations between potential explanatory variables and the *pf*hrp2 gene deletion with a level of statistical significance set at *P* < 0.05.

**Results:**

Of the 684 enrolled symptomatic patients, 391 (57.7%) were female. The majority (87.7%) reported the presence of mosquito breeding sites within the household’s compound, and fever was the most reported symptom (81.6%). The overall prevalence of the *pf*hrp2 gene deletion was 9.2% (95% *CI*: 6.7%–12.1%). The deletion of the *pfhrp2* gene was associated with health zone of origin (*P* = 0.012) and age (*P* = 0.019). Among false-negative *Pf*HRP2-RDT results, only 9.9% were due to *pfhrp2* gene deletion.

**Conclusions:**

*P. falciparum isolates* with *pfhrp2* gene deletions are relatively common among symptomatic patients in Kwilu province. Further investigations are needed to provide enough evidence for policy change. Meanwhile, the use of RDTs targeting *Pf*HRP2 and parasite lactate dehydrogenase (pLDH) antigens could limit the spread of deleted isolates.

**Graphic Abstract:**

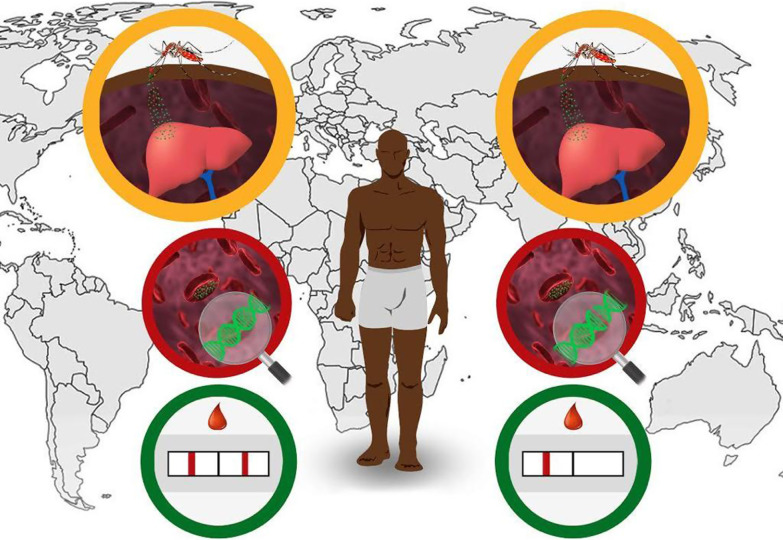

## Background

Malaria remains a global health issue despite progress over the last decade. In 2018, there were an estimated 228 million new malaria cases, including 405 000 deaths [1]. Ninety-two percent of malaria cases and 93% of malaria deaths occurred in Africa. Fifteen countries in sub-Saharan Africa and India carried nearly 80% of the global malaria burden of which Nigeria and the Democratic Republic of the Congo (D.R. Congo) accounted for about 35% [1].

D.R. Congo accounts for 12% of all malaria cases in sub-Saharan Africa [1]. In D.R. Congo, malaria is the leading cause of morbidity and mortality, accounting for more than 40% of all outpatient visits, and for 19% of deaths among under five years children [2]. The high burden of malaria in D.R. Congo can be explained by the fact that nearly the entire population (97%) lives in high-transmission zones where the most common vector encountered is *Anopheles gambiae*, and *Plasmodium falciparum* is the most common species responsible for the majority of severe cases [2, 3].

An important component of malaria control and elimination is appropriate case management, which is based on early and accurate diagnosis. Accurate diagnosis facilitates appropriate and prompt treatment and minimizes the risk of developing drug resistance [1].

The WHO recommends microscopic examination as the gold standard for malaria diagnosis. However, in rural and semi-urban settings where lack of equipment, reagents, trained and skilled personnel, and electricity can prevent this diagnosis method, the use of rapid diagnostic tests (RDTs) offers an alternative for quick and accurate diagnosis [4, 5].

RDTs have become a primary and critical tool for malaria diagnosis in the D.R. Congo as well as in Malaria endemic countries. They accounted for nearly 75% of diagnostic testing among suspected cases in Africa in 2017 [1]. Current RDT kits are designed to detect either *P. falciparum* alone or in combination with other species of human malaria parasites. Three main antigens are detected by malaria RDTs, namely *P. falciparum* histidine rich protein 2 (*Pf*HRP2), parasite lactate dehydrogenase (pLDH), and parasite aldolase (pAldo) [6, 7].

*Pf*HRP2, a *P. falciparum* specific antigen, has the advantage of being highly abundant and heat-stable. *Pf*HRP2-based RDTs can lead to false-positive results in the case of persistent circulating HRP2 antigen as a result of antimalarial treatment, and false-negative results in individuals whose levels of parasitaemia is under the detection threshold of 200 parasites/µl [6, 7].

In the last decade, however, some studies have reported false-negative results among individuals infected with *P. falciparum* parasites presenting a deletion of the *P. falciparum* histidine-rich protein 2 *(pf*hrp*2*) gene. The majority of these studies have also identified co-existing deletions of the *P. falciparum* histidine-rich protein 3 (*pf*hrp*3*) gene, which produces an antigen that shows some cross-reactivity with HRP2 [4, 5, 8–30].

While the WHO recommends not initiating antimalarial treatment without biological evidence, selection of *P. falciparum* isolates with *pfhrp2* gene deletions may occur when only RDT positive patients are treated. The non-treated patients infected by parasites harbouring *pfhrp2* gene deletions will facilitate the spread of *phrp2* deleted strains, jeopardizing progress towards disease control and elimination in low setting countries.

To date, only one study has investigated *pfhrp2*-deleted mutant parasites in D.R. Congo, reporting a country-wide prevalence of 6.4% among children under-five years and providing spatial distribution and population genetics of these deletions [14]. However, this nationwide study could not explore clinical differences between *pfhrp2*-deleted and wild type *P. falciparum* malaria due to limited clinical data and study population (the majority being asymptomatic and under-five), nor was it able to conclude about the relative virulence of *pf*hrp2-deleted parasites.

In order to address the above limitations, we selected Kwilu Province which is classified by the D.R. Congo National Malaria Control Program (NMCP) as a province at high risk of malaria [3]. Kwilu Province is classified in the tropical facies where malaria transmission occurs predominantly during the long rainy season lasting 5 to 8 months, and where the number of infected bites per people per year ranges from 60 to 400 [3]. Using data from a prospective health facility-based cross-sectional study, we aimed to determine the local prevalence of the *pfhrp2* gene deletion among malaria symptomatic patients, and associated clinical, biological, and sociodemographic factors in the Kwilu Province (D.R. Congo). The aim of this study is to contribute to a better characterization of the prevalence and consequences of *pfhrp2* deletions in D.R. Congo by providing relevant regional data to improve malaria management and control.

## Methods

### Study design and setting

**Fig. 1:**
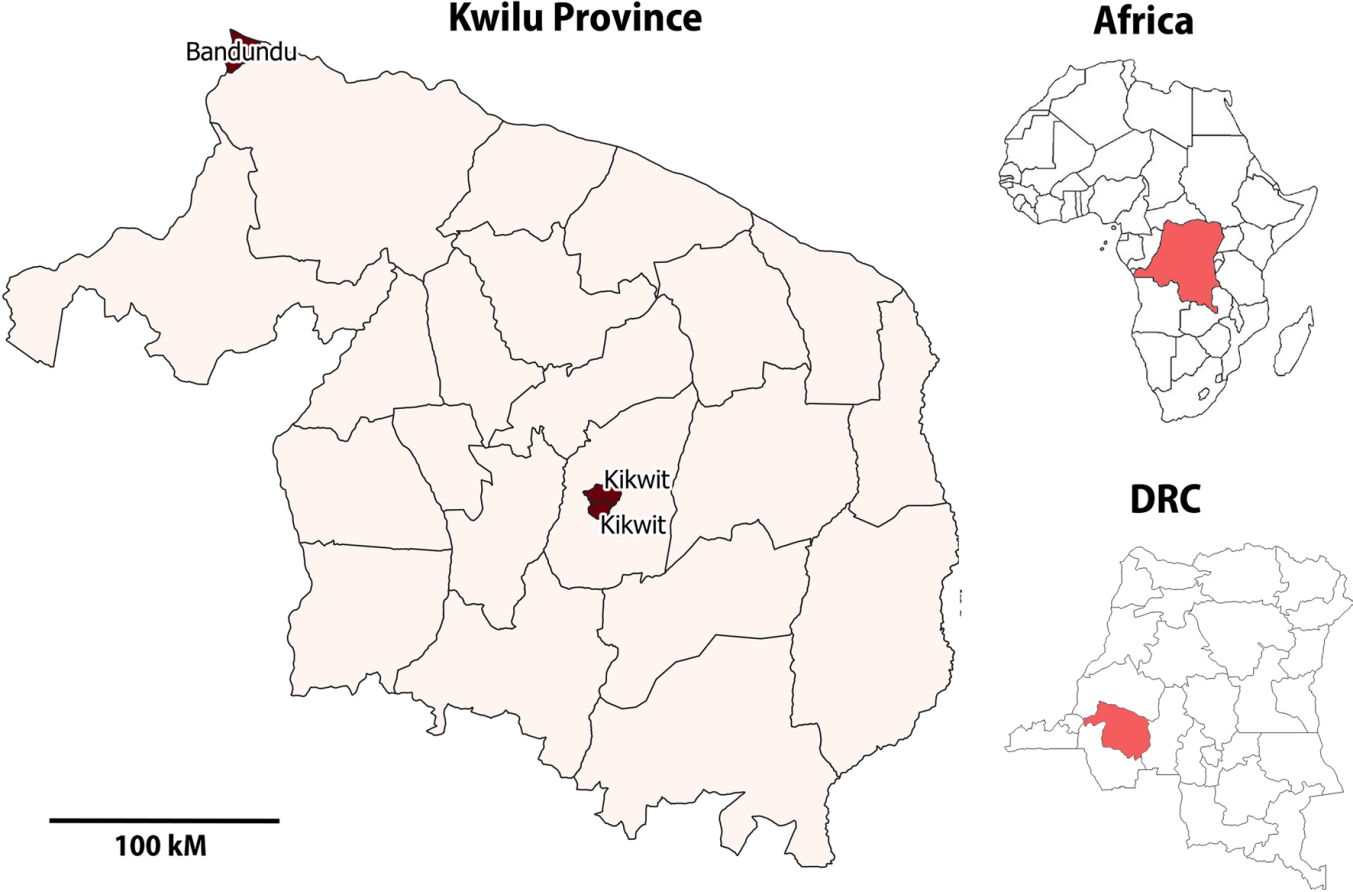
Collection sites

We used secondary data from a prospective health facility-based cross-sectional study conducted on individuals of all ages, seeking healthcare from October to December 2018 in 34 randomly selected health facilities of three health zones in the Kwilu Province (D.R. Congo), Fig. 1.

The Kwilu Province is one of 26 provinces of D.R. Congo with an area of 79 906 km^2^. It is divided into five administrative territories: Bagata (including the city of Bandundu), Bulungu (including the city of Kikwit), Gungu, Idiofa, and Masimanimba [31].

The two selected cities (Bandundu and Kikwit) include three of the 24 health zones of the Kwilu Province [31]. They are the two main cities in the province and bear the highest burden of malaria. *pfhrp2* gene deletions were previously reported in this region [14, 32].

Bandundu, the capital city of the Kwilu Province, is located 400 km from Kinshasa, the capital of D.R. Congo [33]. Bandundu covers an area of 222 km^2^ with a population estimated at 950 683 as of 2015 [33]. It has a tropical wet and dry climate with two seasons. Heavy rainfalls and constant heat characterize the rainy season while fewer rainfalls are recorded during the dry season. The average annual temperature is 26.9 °C [33]. Bandundu City has one semi-urban health zone of the same name and 17 health areas, including 11 urban and six rural.

Kikwit is the second-largest city in Kwilu Province, located in the south-west of D.R. Congo, at 525 km from Kinshasa and 400 km from Bandundu. It is the main economic city of the province and a commercial hub that provides access to diamond-rich regions of Kasaï Province and Angola. Kikwit covers an area of 92 km^2^ with an estimated population of 1 326 068 as of 2016 [34]. The city has a tropical wet and dry climate with a long rainy season from early September through to the end of May and a short dry season from early June to the end of August. Kikwit City has two urban health zones: Kikwit-Nord and Kikwit-Sud.

### Ethics, consent, and permissions

The study was approved by the Kwilu Province Division of Health (N° ADM/707/01/DPS-KLU/CD/JPBK/632/2018), the Kinshasa School of Public Health Ethical Committee (ESP/CE/015/2019) and the School of Tropical Medicine and Global Health Ethical Review Committee.

The study was first explained to all participants, then written and verbal voluntary informed consent was obtained from all study participants including guardian/parents of non-adult participants.

### Study population

The study population included individuals of all ages seeking health care in health facilities located in the three Health Zones of Bandundu (one) and Kikwit (two) cities. Health facilities included General Reference Hospitals, Reference Health Centres, and Health Centres. The smallest selection units were individuals attending these health facilities with symptoms suggestive of malaria. The study included all individuals seeking care in the selected health facilities with symptoms suggestive of malaria such as fever, headaches, malaise; during the study period for whom a laboratory test (*Pf*HRP2-RDT and/or microscopic examination) was performed. Individuals who failed to meet the inclusion criteria or did not consent to participate in the study were excluded.

### Sample size calculation

The minimum number of subjects required to enrol in this study was calculated based on a previously reported proportion of *pfhrp2* gene deletion in the Kwilu Province (3%) and recommendations from WHO for studies on *pfhrp2/3* deletion among symptomatic patients [14, 35]. According to the WHO protocol for estimating *pfhrp2/3* deletion prevalence, for an expected prevalence of 3.2%, at least 370 individuals with *P. falciparum* infection are required per sampling domain [35]. In this study, the sampling domain was the Kwilu province, which included 34 health facilities. The study enrolled a total of 684 patients meeting the inclusion criteria of which 491 were positive for *P. falciparum* using PCR*.*

### Recruitment method

The primary study applied a two-stage random sampling to select health centres. At stage one, 27 health centres were randomly selected among the 62 health centres in the targeted areas. For neighbouring health centres, one health centre was randomly selected out of two. In order to increase the chance of catching individuals not respecting the referral system by directly seeking care in high-level health facilities, four reference health centres and three general reference hospitals from the three health zones were included, bringing the total number of selected health facilities to 34 (27 in Kikwit and seven in Bandundu).

At stage two, individuals attending the selected health facilities with symptoms indicative of malaria were recruited. The lead investigator weighed the number of individuals to recruit per health centre to the average rate of service utilization provided by the National Health Information System.

### Variables

This study used four groups of variables: sociodemographic, malaria prevention, clinical and biological variables. *Plasmodium falciparum* HRP2 gene deletion (*pfhrp2*) was the main outcome variable. Explanatory variables were age, sex, health zones, household size, existence of mosquito breeding sites, LLIN (Long Lasting Insecticidal Net) ownership, use of LLIN, malaria drug intake, malaria clinical features, parasite density, and microscopy result.

### Data collection method

Potential participants were introduced to the study by a research assistant. After securing consent/assent from the subjects or their guardians, socio-demographic, malaria prevention and treatment practices, and clinical variables were collected using a pre-tested structured questionnaire. Patients’ medical records were used to collect data from the physician’s or health officer’s clinical examination.

Heel or finger-prick blood was collected from each individual. Samples for microscopy were prepared using two drops of blood. Then 50 µl of blood were applied on *Pf*HRP2-RDT, and a few drops were spotted onto Whatman filter paper to prepare dried blood spots (DBS).

The membranes of spent *Pf*HRP2-RDT cassettes and the DBSs were individually stored in plastic bags, sealed with a desiccant at room temperature before being shipped to the Institute of Tropical Medicine in Nagasaki (NEKKEN) where they were refrigerated at 4 °C.

### Malaria RDT screening

The CareStart™ Malaria *Pf* (HRP2) Ag RDT (Access Bio, Inc., Somerset, New Jersey, USA) was used for the qualitative detection of malaria histidine-rich protein 2 in the whole blood according to the manufacturer’s instructions [ACCESSBIO, 2018, Somerset, New Jersey, USA]).

The test membrane strip is pre-coated with a *P. falciparum* HRP2 specific monoclonal antibody as a single line across the test strip. The reported panel detection score is 91.0% at 200 parasites/µl with a false positive rate of 0.9% [36, 37]

### Microscopic diagnosis of malaria

A team of four medical technologists read the slides in the laboratories of health facilities where samples were collected. When a health facility did not have the necessary equipment to perform the examination, slides were read at the nearest laboratory possessing adequate equipment. For quality assurance, one expert microscopist randomly selected positive and negative slides to cross-check results. In the case results were not concordant, another reading was performed. About five percent of slides went through another quality control in the vector control laboratory of the Kinshasa School of Public Health.

Thick and thin smears were made on the same slide. The part of the slide containing the thin smear was fixed with methanol and dried. Then the whole slide was stained with 10% Giemsa’s solution for ten minutes and finally washed off with distilled water and air-dried. Stained smears were examined under a microscope for malaria parasite identification. For positive slides, malaria parasites were counted against 200 white blood cells (WBC), and parasite density was calculated based on a total of 8000 WBC/µl using the following formula: (Number of Parasites counted × 8000)/Number of counted WBC.

Parasite density calculation was immediately performed when 100 parasites were counted against 200 WBC. However, in the case that fewer than 100 parasites were counted against 200 WBC, the count continued until 500 WBC.

### Extraction of parasite DNA

Genomic DNA was extracted from membranes of spent *Pf*HRP2-RDT cassettes and DBS using the QIAGEN QIAmp®DNA extraction kit (company, city, country) according to the manufacturer’s instructions. We also adapted a previously described method to recover DNA from 197 spent RDTs membranes from Bandundu Health Zone [38].

### Detection of *P. falciparum* infection & pfhrp2 gene deletion

**Fig. 2:**
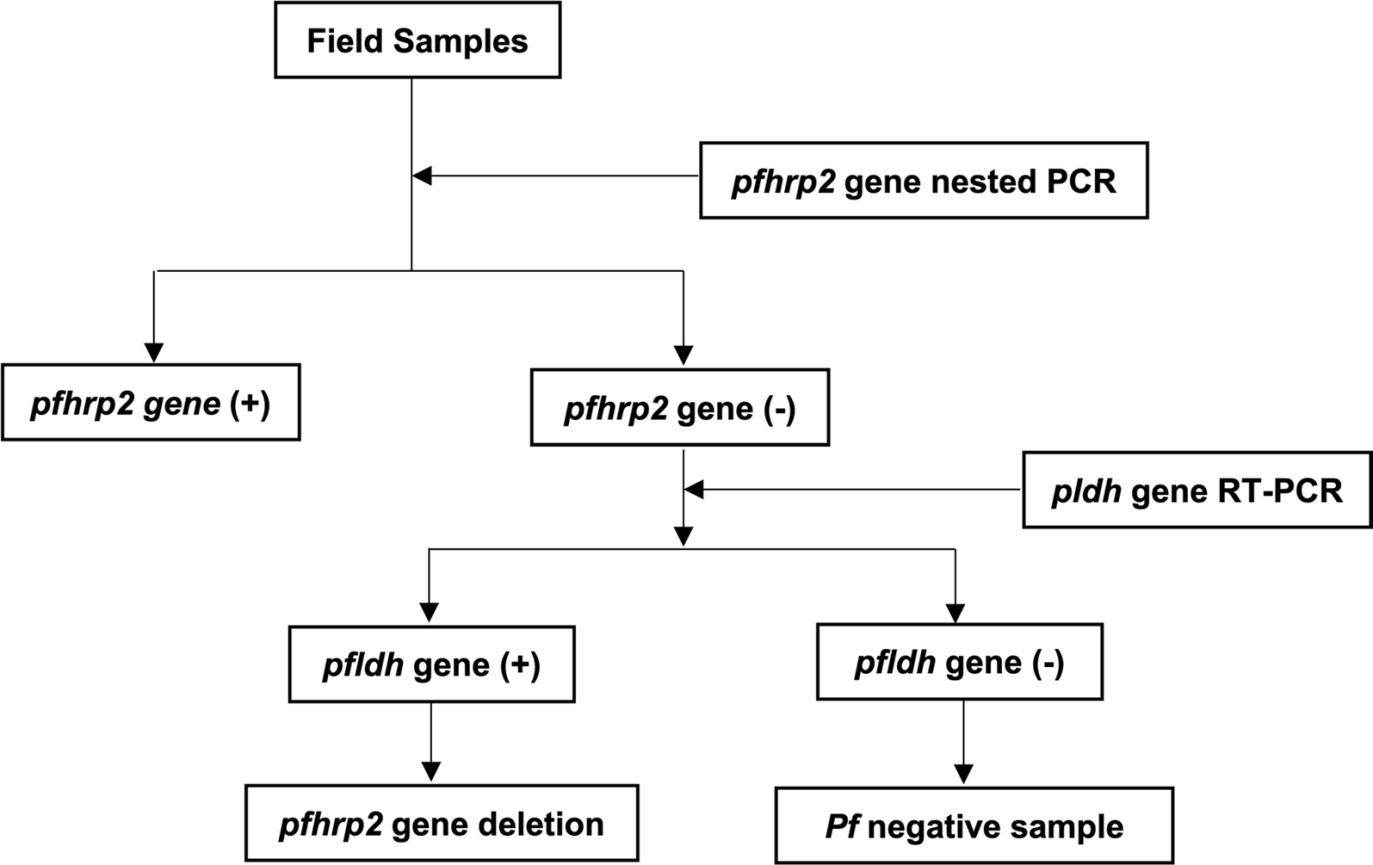
*pfhrp2* gene deletion testing pipeline

To confirm *P. falciparum* infection, we designed specific primers targeting a 226 base pair region of the *P. falciparum* lactate dehydrogenase (*pfldh*) gene and performed a real-time PCR assay (Table 1). This assay was also used to ensure there was sufficient parasite DNA quantity in the samples to discriminate *P. falciparum* negative samples from samples with *pfhrp2* gene deletion, as shown in Fig. 2.

**Table 1:** Primer sequences and PCR conditions for *pfhrp2 and pfldh* genes amplification

Targeted gene	Primer sequence (5′ → 3′)	Reaction component	Cycling condition	LOD (ng/µl)
*pfhrp2* Exon 1–2, PF3D7_0831800	Outer For: GGTTTCCTTCTCAAAAAATAAAG Rev: TCTACATGTGCTTGAGTTTCG	One *Taq* 2 × Master Mix with standard buffer: 12.5 µl 10 µmol/L forward primer: 1 µl 10 µmol/L reverse primer: 1 µl Nuclease free water: 7.5 µl DNA template: 3 µl (gDNA or 5 × diluted outer PCR product 25 µl reaction volume	95 °C/5 min; 40 cycles of 95 °C/30 s, 55 °C/30 s, 68 °C/30 s 68 °C/5 min 4 °C–∞	10^–5^
	Inner For: GTATTATCCGCTGCCGTTTTTGCC Rev: CTACACAAGTTATTATTAAATGCGGAA		95 °C/5 min; 40 cycles of 95 °C/30 s, 62 °C/30 s, 68 °C/30 s 68 °C/5 min 4 °C–∞	
*pfldh* (qPCR)	For: ACGATTTGGCTGGAGCAG Rev: GGAACACCTGAATGTTGATG	PowerUp™ SYBR ™ Green Master Mix (2 ×): 12.5 µl 10 µmol/L forward primer: 0.5 µl 10 µmol/L reverse primer: 0.5 µl Nuclease free water: 6.5 µl DNA template: 2–4 µl 22–24 µl reaction volume	50 °C/2 min; 95 °C/2 min 45 cycles of 95 °C/15 s, 62 °C/1 min, 95 °C/30 s, 60 °C/15 s	10^–4^

*LOD* Lower limit of detection, *qPCR* Quantitative or real-time PCR

Samples were duplicated and loaded in 96-wells plates along with serially diluted positive controls (gDNA from in vitro cultured *P. falciparum* strain 3D7) (1 ng/µl, 0.1 ng/µl, 0.01 ng/µl, 0.001 ng/µl), as well as negative controls containing DNA from blood spots prepared from known malaria negative individuals. We repeated the assay for all discordant duplicates and counted three consistent results as the final result.

For detection of the *pfhrp2* gene, we performed a nested PCR assay using primers targeting a 228 base pair fragment spanning exon 1, the intron, and a portion of exon 2 of *pfhrp2* as previously described (Table 1) [9]. We used a lower elongation temperature (68 °C) to improve PCR sensitivity, *pfhrp2* being AT-rich, and increased the number of cycles to 40. We used genomic DNA from Dd2 (*pfhrp2* negative) and 3D7 (*pfhrp2* positive) as controls for all assays.

We repeated the nested PCR for all negative results. In the case of discordant results, we performed the amplification a third time and counted two consistent results as the final result.

Reaction components for both real-time and nested PCR are summarized in Table 1.

### PCR product resolution by agarose gel electrophoresis

PCR amplicons were separated by electrophoresis on a 2% agarose gel stained with Gel Red® Nucleic Acid Stain 10 000 × in water. A total of 12 µl of PCR amplicons (6 µl) and loading dye (6 µl) were loaded onto the gel, which was run for 35 min at 100 V and observed under UV light. A 500 µg/ml Gene Ruler 100 bp DNA Ladder (BioLabs®inc, Ipswich, USA) was loaded onto the same gel to determine the sizes of the resolved fragments.

### Statistical analyses

Data were entered and analyzed using STATA15 (StataCorp LLC, Lakeway, Texas, USA). Tables have been used to describe categorical variables. Continuous variables have been summarized using median and interquartile ranges. Proportions have been used to summarize categorical variables.

Fischer’s exact test (for categorical variables) and the Kruskal–Wallis test (for non-normally distributed continuous variables) were applied to look for associations between potential explanatory variables and the main outcome (*pf*hrp2 gene deletion). We computed the 95% Confidence Interval (95% CI) for the prevalence of *pfhrp2* gene deletion. We considered a *P*-value less than 0.05 statistically significant.

## Results

### Socio-demographic characteristics

Of the 684 symptomatic patients who participated in the study; 391 (57.7%) were female, and 287 (42.3%) were male. Kikwit-Nord Health Zone accounted for the majority of patients 362 (52.9%) while Bandundu and Kikwit-Sud Health Zones accounted for 197 (28.8%) and 125 (18.3%) patients, respectively.

The median age was nine years, with an interquartile range from 3 to 26 years old. Children under five years old represented 36% of enrolled patients. The median size of household was six, with an interquartile range from five to six. Table 2 summarizes socio-demographic characteristics.

**Table 2:** Socio-demographic characteristics, household environment and malaria prevention and treatment practices

Characteristic	*n*	%	Median	IQR
Sex (*n* = 678)				
Female	391	57.7		
Male	287	42.3		
Health Zone (*n* = 684)				
Bandundu	197	28.8		
Kikwit-Nord	362	52.9		
Kikwit-Sud	125	18.3		
Age in years (*n* = 680)			9	3–26
< 5	245	36.0		
> 5	435	64.0		
Household Size (*n* = 684)			6	5–8
Household environment (*n* = 684)				
Mosquito breeding sites	600	87.7		
LLIN ownership	439	64.2		
LLIN utilisation	340	49.7		
Malaria prevention and treatment practices				
Prior drug intake (*n* = 684)	220	32.2		
Malaria drug taken (*n* = 220)				
Quinine	89	13.0		
Sulfadoxine–pyrimethamine	42	6.1		
Artemether–lumefantrine	33	4.8		
Artesunate	14	2.0		
Arteether	13	1.9		
Artemether	12	1.8		
Arteether-artemotil	10	1.5		
Amodiaquine	4	0.6		
Artesunate-sulfadoxine-pyrimethamine	2	0.3		
Plant extract	1	0.1		

For the variable sex, less than 1% information is missing (six entries). For the variable age, less than 1% information is missing (four entries)

Malaria drug taken includes only individuals who reported malaria drug intake at least one week before the survey

*IQR* Inter quartile range, *LLIN* Long lasting insecticidal net

### Household environment, malaria prevention, and treatment practices

The majority (87.7%) of patients/patients’ guardian reported the presence of mosquito breeding sites within the compound where the household was located. Two-thirds of household (64.2%) owned a mosquito bednet, while 49.7% of patients spent the night before the interview under a mosquito bed net. One-third (220) of patients reported prior malaria drug intake. Quinine (13%), sulfadoxine-pyrimethamine (6.1%), artemether-lumefantrine (4.8%), artesunate (2.0%), arteether (1.9%), artemether (1.8%) were the common drugs taken as illustrated in Table 2.

### Clinical and biological features of symptomatic patients

At admission, the body temperature of patients ranged from 37 °C to 38 °C (median temperature of 37.5 °C). The common findings of the clinical examination were: A history of fever the last 72 h (81.6%), headaches (41.8%), vomiting (31.4%), rigor (22.8%), fatigue (22.1%) and abdominal pain (20.2%). Among the 235 patients with a microscopy positive result, malaria parasite density ranged from 64 parasites /µl to 7200 parasites /µl with a median parasite density of 880 parasites/µl. Table 3 summarizes the distribution of clinical and biological features.

**Table 3:** Clinical and biological features of symptomatic patients

Characteristic	*n*	%	Median	IQR
Clinical features (*n* = 684)				
History of fever	558	81.6		
Headache	286	41.8		
Vomiting	215	31.4		
Rigor	156	22.8		
Fatigue	151	22.1		
Abdominal pain	138	20.2		
Joint Pain	58	8.5		
Anorexia	42	6.1		
Neckache	34	5.0		
Diarrhoea	32	4.7		
Pallor	13	1.9		
Convulsions	12	1.8		
Splenomegaly	5	0.7		
Impaired consciousness	3	0.4		
Temperature at admission (°C)			37.5	37–38
Biological features (*n* = 235)				
Parasite density (parasites /µl)			880	64–7200
P arasite density accounts for only microscopy positive samples				

*IQR* Inter quartile rRange, *LLIN* Long lasting insecticidal net

### Comparison of RDT, PCR and microscopy

Among patients with a negative *Pf*HRP2-RDT result, 63.9% were ‘false negatives’ compared to PCR. The sensitivity and specificity of RDTs with reference to PCR were 71.3% (325/456) and 42.4% (74/173), respectively. Using microscopy as the gold standard, the proportion of false-negative RDT result dropped to 26.3%. The microscopy-determined sensitivity and specificity of RDTs were 74.3% (156/210) and 36.0% (151/419), respectively (Table 4).

**Table 4:** Comparison of HRP2-RDT, PCR, and microscopy

HRP2-RDT (*n* = 629)	PCR				Microscopy			
	Negative		Positive		Negative		Positive	
	*n*	%	*n*	%	*n*	%	*n*	%
Negative	74	36.1	131	63.9	151	73.7	54	26.3
Positive	99	23.3	325	76.7	268	63.2	156	36.8

*HRP2-RDT* Histidin Rich Protein 2 based rapid diagnostic test, *PCR* Polymerase chain reaction

### Proportion of pfhrp2 gene deletion in false-negative PfHRP2-RDT

Using PCR as the gold standard, there were 131 false-negative *Pf*HRP2-RDT results of which only 9.9% were due to *pfhrp2* gene deletion, with a 95% *CI* ranging from 5.4% to 16.4%. The mean parasitaemia of the 131 RDT false-negative infections was 2447 parasites per µl. The theoretical limit of detection of *Pf*HRP2-RDT is 200 parasites per ul of blood. Of 155 samples with parasite densities of over 200 parasites per ul as determined by microscopy, 30 were negative by RDT (19%). Of these, only one harboured a deletion at the PfHRP2 locus.

### Prevalence of pfhrp2 gene deletion among all symptomatic PfPCR confirmed cases

The overall prevalence of *pfhrp2* gene deletion among *Pf*PCR confirmed symptomatic cases was 9.2%. The highest prevalence was found in Bandundu Health Zone (15.7%), followed by Kikwit-Sud Heath Zone (9.6%) and Kikwit-Nord Health (6.2%). Table 5 shows the distribution of *pf*hrp2 gene deletion among PCR confirmed cases across Heath Zones.

**Table 5:** Frequency of *pfhrp2* gene deletion among all symptomatic *Pf*PCR confirmed cases

Health Zones	Subjects	*Plasmodium falciparum* infection		*pf*hrp2 gene deletion	
		*n*	Prevalence (95% *CI*)	*n*	Prevalence (95% *CI*)
Overall	684	491	71.8 (68.2–75.1)	45	9.2 (6.7–12.1)
Bandundu	197	121	61.4 (54.2–68.2)	19	15.7 (9.7–23.4)
Kikwit-Nord	362	276	76.2 (71.5–80.5)	17	6.2 (3.6–9.7)
Kikwit-Sud	125	94	75.2 (66.7–82.5)	9	9.6 (4.5–17.4)

*PCR* Polymerase chain reaction, *CI* Confidential interval

### Socio-demographic, malaria prevention, biological features, and pfhrp2 gene deletion

As shown in Table 6, there was no statistically significant difference in *pfhrp2* gene deletion status between males and females (8.5% vs 9.8%). Conversely, there was a statistically significant difference in *pf*hrp2 gene deletions status in Bandundu health zone compared to Kikwit-Nord and Kikwit-Sud health zones (*P* = 0.012). There was a trend towards *pfhrp2* gene deletion in older compared to younger patients, but this was not statistically significant (*P* = 0.079). However, when analyzing age as a continuous variable, the median age among *pfhrp2* gene deleted patients was higher than the median age among *pfhrp2* gene non-deleted patients (18 years vs 7 years). The Kruskal Wallis test showed strong evidence suggesting that the distributions of age differed by *pf*hrp2 gene deletion status (*P* = 0.019). Similarly, there was a trend towards *pfhrp2* gene deletion among negative microscopy results, but weak evidence supported this finding (*P* = 0.079).

**Table 6:** Socio-demographic, malaria prevention, biological features and *pf*hrp2 gene deletion

Characteristic	*pfhrp2* gene deleted				*pfhrp2* gene non-deleted				*P*-value
	*n*	%	Median	IQR	*n*	%	Median	IQR	
Sex (*n* = 487)									
Female	27	9.8			248	90.2			0.640
Male	18	8.5			194	91.5			
Health Zone (*n* = 491)									
Bandundu	19	15.7			102	84.3			**0.012**
Kikwit-Nord	17	6.2			259	93.8			
Kikwit-Sud	9	9.6			85	90.4			
Age in years (*n* = 488)			18	4.9–30			7	2.9–19	0.019*
< 5	12	6.3			179	93.7			0.079
> 5	33	11.1			264	88.9			
Household size (*n* = 491)			7	6–8			6	5–8	0.388*
Mosquito breeding sites (*n* = 491)									
Absent	8	14.0			49	86.0			0.217
Present	37	8.5			397	91.5			
LLIN ownership (*n* = 491)									
No	16	9.0			161	91.0			> 0.999
Yes	29	9.2			285	90.8			
LLIN utilization (*n* = 491)	24	9.5			228	90.5			0.876
No	21	8.8			218	91.2			
Yes									
Prior drug intake (*n* = 491)									
No	29	9.0			295	91.0			0.869
Yes	16	9.6			151	90.4			
Microscopy (*n* = 491)									
Negative	33	11.1			265	88.9			0.079
Positive	12	6.2			181	93.8			
Parasite density (*n* = 491)			4680	264–14 800			1200	112–8080	0.3771*
Clinical features (*n* = 491)									
Temperature at admission (°C)			37.5	36.4–38.6			37.8	37–38	0.345*
History of fever	34	8.4			371	91.6			0.217
Headache	19	9.4			183	90.6			0.875
Vomiting	11	6.6			156	93.4			0.187
Rigor	5	4.4			108	95.6			0.061
Fatigue	9	8.3			99	91.7			0.851
Abdominal pain	5	4.8			100	95.2			0.087
Joint pain	3	7.7			36	92.3			1.000
Anorexia	3	9.4			29	90.6			1.000

Kruskal Wallis test (*) has been used to compare distributions for continuous variables (parasite density, age, and household size), Fischer exact test has been used for categorical variables

*Pfhrp2*: *Plasmodium falciparum* Histidin Rich Protein 2 gene, *IQR* Inter quartile range, *LLIN* Long lasting insecticidal net

### Clinical features and pfhrp2 gene deletion

We found more malaria signs and symptoms among patients infected with parasites not harbouring *pfhrp2* gene deletion. However, the difference was not statistically significant (Table 6).

## Discussion

More females participated in this study than males (57% vs 42.3%). This finding is in keeping with results from the D.R. Congo 2013–2014 Demographic and Health Survey(DHS) which reported a sex ratio slightly in favour of females [39].

Surprisingly, children under-five represented 36% of participants. This is in contrast with what might be expected since under-five children are known to be at higher risk of contracting malaria. Despite the burden of malaria in Kwilu Province, there is a seasonal trend in transmission, and immunity is acquired later in life, around 10 years [3]. Late acquisition of immunity can explain the higher proportion of participants aged more than five years, seeking treatment for symptoms suggestive of malaria and thus enrolled in this study. The median household size of six is similar to findings from the 2013–2014 DHS which reported of median size of 5.7 [39].

There were mosquito breeding sites near households of the majority of participants (87.7%). The survey was conducted during the rainy season, which is characterized by the formation of breeding sites, especially in rural and semi-urban areas with limited public facilities.

The use of mosquito bednets was the primary means of bite-prevention. Two-thirds of households (64.2%) owned a bednet, and only 49.7% of participants spent the night before the interview under a mosquito bednet. These results are lower than the previous report from the DHS in Kwilu Province. In 2014, 87% of households surveyed in the Kwilu province possessed a bednet, and 69.4% of participants slept under a bednet the night before the interview [39]. Back then, an extensive mosquito bednet distribution campaign was implemented with a higher rate of implementation, especially in Kwilu. However, Mwandagalirwa et al*.* recently reported consistent data (72% ownership vs 45% use) in health zones of Kinshasa province, neighbouring Kwilu province [40]. A low coverage during bednet distribution campaigns can explain the lower proportion of household possessing bed net. Also, bednet usage is known to be higher among under-five children and falls progressively to as low as 34% by the early twenties [40]. Subsequently, the majority of participants (64%) being over five years may account for the majority of participants not using bednets.

Among participants who reported prior malaria drug intake, 13% took quinine at least one week before the survey. This finding highlights the poor compliance with the WHO guidelines for the treatment of malaria in the study areas as well as challenges associated with the use of injectable artesunate in areas where quinine is cheaper, easy to use and available.

As might be expected, 81.6% of participants reported a history of fever. In many cases, fever is suggestive of malaria but is also reported in several febrile illnesses prevailing in tropical areas. Without a reliable diagnostic tool, a syndromic approach often leads to overtreatment, especially among people living in low to moderate malaria transmission areas [41]. A recent analysis of household survey data from 24 Sub-Saharan Africa countries between 2006 and 2014 showed that 35.7% of all fevers reported by participants were accompanied by malaria infection evidence, but only 10% of these fevers were attributable to malaria [42]. Non-malarial febrile illnesses (NFMI) can coincide with malaria infection, and may lead to over-diagnosis of malaria and underestimation of the burden of associated NFMI [42].

Only 9.9% of false-negative *Pf*HRP2-RDT results involved parasites with *pfhrp2* gene deletions. A similar result (10.6%) has been reported in Nigeria, [24]. Conversely, Wurtz et al. (2013), in Senegal reported a lower proportion (2.4%) while Amoah *et* al. (2016), in Ghana reported a higher proportions of 23% [4, 5, 17].

This weak proportion shows that *pfhrp2* gene deletion is not a major cause of false-negative *Pf*HRP2-RDT results. Several reasons may explain a false-negative *Pf*HRP2-RDT result such as poor quality of the test, inappropriate manipulation and interpretation, low parasite density, excess of circulating parasite antigens creating a prozone-like effect, and genetic polymorphisms in the target antigen [6, 23, 43, 44].

Even though the proportion of *Pf*HRP2-RDT false-negative results due to *pfhrp2* gene deletion has surpassed the 5% threshold set by the WHO, requiring a subnational change in malaria RDTs, the required number of *P. falciparum* isolates (37) to include per health facility in the sampling domain was not reached. Therefore, further investigations are needed to provide enough evidence for policy change.

The overall prevalence of *P. falciparum* isolates with *pfhrp2* gene deletion was 9.2%. This prevalence is higher than the previously reported national prevalence of 6.4% and the local prevalence of 3% [14]. The present study exclusively enrolled subjects with symptoms suggestive of malaria and thus more likely to be infected while the previous one included more asymptomatic subjects. Secondly, Kwilu Province is located in a low to moderate transmission area where immunity is acquired later in life. This condition of reduced host immunity is favourable to infection by parasites harbouring *pfhrp2* gene deletion, which can survive and spread [18]. Thirdly, the prevalence of parasites harbouring *pfhrp2* gene deletion has been shown to be higher in low to moderate transmission area in the beginning of the rainy season, which is the case for the present study [45].

A similar prevalence has been reported in Eritrea (9.7%) and Kenya (9%) [13, 46]. However, lower prevalence has been reported in Senegal (2.4%), Mozambique (1,4%) and higher prevalence in Nigeria (17%), Ghana (36%) and Zambia (37.5%) [4, 15–17, 24, 25]. The difference in study design and methodology used for deletion confirmation (nested PCR vs qPCR of a single-copy gene) could explain the discrepancy. Publications using nested PCR for confirmation of deletion tend to overestimate the prevalence while amplification of a single-copy gene by real-time PCR is recommended for appropriate deletion call [47, 48].

The first protocol concerning *phrp2/3* deletions was released in 2014 [49]. Subsequently, the WHO released a second protocol in 2018 for estimating *phrp2/3* deletions among symptomatic patients [35]. Finally, Parr et al. revised the existing protocols and released a streamlined protocol taking into account challenges faced by previous authors [48].

There was a statistically significant difference in *pfhrp2* gene deletion prevalence in Bandundu health zone compared to Kikwit-Nord and Kikwit-Sud health zones (*P* = 0.012). Variations in *pfhrp2* gene deletion status within regions and countries have been previously reported and depend on several factors including level of transmission and magnitude of *Pf*HRP2-RDT use [10, 12, 20, 50]. Further analysis of population genetics may clarify this finding.

There was a trend towards *pfhrp2* gene deletion in older compared to younger (under-five) patients, but this was not statistically significant (*P* = 0.079). However, when analyzing age as a continuous variable, the Kruskal Wallis test showed strong evidence suggesting that the distributions of age differed by *pf*hrp2 gene deletion status (*P* = 0.019). This may suggest that the risk of being infected with parasites harboring *pfhrp2* deletion increases with age while traditionally under-5 years old are at high risk of contracting malaria compared to the older group.

Similarly, there was a trend towards *pfhrp2* gene deletion among negative microscopy results, but weak evidence supported this finding (*P* = 0.079). Microscopic examination is based on morphological aspects of the parasite and might not be influenced by genetic traits such as gene deletions. Even considering that *Pf*HRP2 is known to be involved in the formation of hemozoin, this is not the only morphological feature allowing parasite detection by microscopic examination.

We found more malaria signs and symptoms among patients infected with parasites not harbouring *pf*hrp2 gene deletions. However, the difference was not statistically significant. This study could only identify 45 *P. falciparum* isolates with *pfhrp2* gene deletions, and a larger sample size may be required to detect differences and provide evidence of association.

This study is the first to provide the local prevalence of *P. falciparum* isolates with *pfhrp2/3* gene deletion among symptomatic patients in this region. The availability of clinical, biological, and sociodemographic data allowed exploration of differences between infection by *pfhrp2*-deleted and wild-type *P. falciparum* parasites. However, the limited sample size precluded identification of predictors of *pfhrp2* gene deletion and did not allow us to make solid conclusions regarding differences in pathology between *pfhrp2* deleted and wild-type parasites.

The selection of study sites based on known burden of malaria and sociodemographic characteristics may have introduced a selection bias making the sample not representative of the whole province within which the level of transmission varies. A health facility-based design is undoubtedly the best choice to recruit symptomatic patients, but the low service utilization rate and insufficient public subsidies allocated to malaria management may have prevented some subjects with the characteristic of interest to attend health facilities and thus to be enrolled in the study.

The D.R. Congo National Malaria Control Program has adopted a five year (2016–2020) strategic plan with goals including the diagnosis of at least 80% of fever cases and the treatment of all positive diagnosed cases with ACT. Increased service utilization, the significant use of *Pf*HRP2 based RDTs as primary diagnostic tools and co-infections with non-malaria febrile illnesses could lead to the initiation of a selective treatment favoring *pfhrp2* deleted parasites. There is a need, therefore, to establish a surveillance system for *pfhrp2* deleted mutants as a part of malaria control programs. Such a surveillance system should be strengthened with reliable diagnostic tools such as molecular point of care testing to ensure efficient and evidence-based allocation of resources to disease control programs.

In routine practice, clinicians should investigate other febrile illnesses despite a positive RDT result to minimize failure in disease management.

## Conclusions

We found a local prevalence of 9.2% of *P. falciparum* isolates with a *pfhrp2* gene deletion among symptomatic patients. These isolates explained only 9.9% of *Pf*HRP2-RDT false-negative results, suggesting that factors other than *pfhrp2* gene deletion are of significant importance in the false-negativity rates of *Pf*HRP2-based RDTs. Even though the proportion of false-negative *Pf*HRP2-RDT results due to the *pfhrp2* deletion has surpassed the 5% threshold set by the WHO for a subnational change in malaria RDTs, further regional investigations with appropriate sampling are needed to provide enough evidence for policy change. Meanwhile, the use of RDTs targeting *Pf*HRP2 and pLDH antigens could limit the spread of deleted isolates.

## Data Availability

The datasets used and/or analysed during this study are available from the corresponding author on reasonable request.

## Abbreviations

°C: Degree centigrade
ACT: Artemisinin-based Combination Therapy
Ag: Antigen
CAID: Cellules d’Analyses des Indicateurs de Developpement/Unit for development indicators analysis
D.R. Congo: Democratic Republic of Congo
DBS: Dried Blood Spot
DNA: Deoxyribonucleic Acid
ELISA: Enzyme-linked Immunosorbent Assay
g/dL: Gram per deciliter
HIV/AIDS: Human Immunodeficiency Virus/Acquired Immunodeficiency Syndrome
IQR: Inter Quartile Range
kDa: KiloDalton
LLIN: Long Lasting Insecticidal Net
LOD: Limit of Detection
mmHg: Millimeter mercury
mmol/L: Millimole per liter
ng: Nanogram
NMFI: Non-Malaria Febrile Illness
pAldo: Parasite Aldolase
PCR: Polymerase Chain Reaction
*Pf*HRP1: *Plasmodium falciparum* Histidine Rich Protein 1
*pfhrp2*: *Plasmodium falciparum* Histidine Rich Protein 2 gene
*Pf*HRP2: *Plasmodium falciparum* Histidine Rich Protein 2
*pf*HRP2-RDT: *Plasmodium falciparum* Histidine Rich Protein 2 based Rapid Diagnostic Test
*Pf*HRP3: *Plasmodium falciparum* Histidine Rich Protein 3
*pfldh*: *Plasmodium falciparum lactate dehydrogenase gene*
*PfPCR*: *Plasmodium falciparum* Polymerase Chain Reaction
pLDH: Parasite Lactate Dehydrogenase
PNLP: Programme National de Lutte contre le Paludisme / National Malaria Control Programme
QBC: Quantitative Buffy Coat
qPCR: Quantitative Polymerase Chain Reaction
RBC: Red Blood Cell
RDT: Rapid Diagnostic Test
SP: Sulfadoxine-Pyrimethamine
USAID: United States Agency for International Development
WBC: White Blood Cell
WHO: World Health Organization
µl: Microliter
